# Impact of ESKAPE Pathogens on Bacteremia: A Three-Year Surveillance Study at a Major Hospital in Southern Italy

**DOI:** 10.3390/antibiotics13090901

**Published:** 2024-09-21

**Authors:** Mariagrazia De Prisco, Roberta Manente, Biagio Santella, Enrica Serretiello, Federica Dell’Annunziata, Emanuela Santoro, Francesca F. Bernardi, Chiara D’Amore, Alessandro Perrella, Pasquale Pagliano, Giovanni Boccia, Gianluigi Franci, Veronica Folliero

**Affiliations:** 1 Clinical Pathology and Microbiology Unit, San Giovanni di Dio and Ruggi D’Aragona University Hospital, 84131 Salerno, Italy; depriscomariagrazia22@gmail.com (M.D.P.); manente392@gmail.com (R.M.); enrica.serretiello@unicampania.it (E.S.); gboccia@unisa.it (G.B.); 2 U.O.C. of Virology and Microbiology, University Hospital “Luigi Vanvitelli”, 80138 Naples, Italy; federica.dellannunziata@unicampania.it; 3 Department of Medicine, Surgery and Dentistry ‘’Scuola Medica Salernitana’’, University of Salerno, 84081 Salerno, Italy; bi.santella@gmail.com (B.S.); esantoro@unisa.it (E.S.); ppagliano@unisa.it (P.P.); 4 U.O.D. Tutela della Salute e il Coordinamento del Sistema Sanitario Regionale—Regione Campania, 80143 Naples, Italy; bernardi.francesca.futura@gmail.com; 5 U.O.C Clinica Malattie Infettive, Azienda Ospedaliera Universitaria, San Giovanni di Dio and Ruggi D’Aragona University Hospital, 84131 Salerno, Italy; chr.damore@gmail.com; 6 Unit Emerging Infectious Disease, Ospedali dei Colli, P.O. D. Cotugno, 80131 Naples, Italy; alessandro.perrella@ospedalideicolli.it; 7 U.O.C Hospital and Epidemiological Hygiene, San Giovanni di Dio and Ruggi D’Aragona University Hospital, 84131 Salerno, Italy

**Keywords:** bloodstream infections, antimicrobial resistance, ESKAPE, antibiotics

## Abstract

Background/Objectives: ESKAPE pathogens (*Enterococcus faecium*, *Staphylococcus aureus*, *Klebsiella pneumoniae*, *Acinetobacter baumannii*, *Pseudomonas aeruginosa*, and *Enterobacter* spp.) pose a serious public health threat as they are resistant to multiple antimicrobial agents. Bloodstream infections (BSIs) caused by ESKAPE bacteria have high mortality rates due to the limited availability of effective antimicrobials. This study aimed to evaluate the prevalence and susceptibility of ESKAPE pathogens causing BSIs over three years in a large tertiary hospital in Salerno. Methods: Conducted at the Clinical Microbiology Laboratory of San Giovanni di Dio e ‘‘Ruggi D’Aragona’’ Hospital from January 2020 to December 2022, blood culture samples from different departments were incubated in the BD BACTEC™ system for 5 days. Species identification was performed using MALDI-TOF MS, and antimicrobial resistance patterns were determined by the VITEK2 system. Results: Out of 3197 species isolated from positive blood cultures, 38.7% were ESKAPE bacteria. Of these, 59.9% were found in blood culture samples taken from men, and the most affected age group was those aged >60 years. (70.6%). *Staphylococcus aureus* was the main BSI pathogen (26.3%), followed by *Klebsiella pneumoniae* (15.8%). Significant resistance rates were found, including 35% of *Staphylococcus aureus* being resistant to oxacillin and over 90% of *Acinetobacter baumannii* being resistant to carbapenems. Conclusions: These results highlight the urgent need for antimicrobial stewardship programs to prevent incurable infections.

## 1. Introduction

Bloodstream infections (BSIs) account for 15% of all nosocomial infections and are among the leading causes of mortality in hospitalized patients, representing a serious international health problem [1,2]. In Europe and North America, BSIs rank among the top seven causes of death and contribute to approximately a quarter of a million fatalities each year [3,4,5]. In Italy, 3056 cases of BSI were reported in 2022. In 2021, there were 2396 reported cases; this number has since increased, probably due to the large use of antibiotics during the COVID-19 pandemic period [6]. Most BSIs were caused by *Klebsiella pneumoniae*, with 95.2% of these isolates producing the carbapenemase (KPC) enzyme [7]. Antimicrobial resistance (AMR) poses a serious potential threat to global health, leading to high mortality and morbidity rates. Each year, antibiotic-resistant infections result in approximately 33.000 deaths in Europe and 700,000 worldwide. Projections indicate that by 2050, these infections could cause up to 10 million deaths per year worldwide [8]. In Italy, the situation is particularly concerning, as the country ranks among the highest in Europe for both antimicrobial consumption and the prevalence of resistant bacterial strains [9]. The rapid spread of AMR presents profound clinical and economic challenges, necessitating urgent international intervention [10]. The most common bacteria isolated in BSIs are the ESKAPE bacteria, which are a major concern due to their multidrug resistance (MDR) profiles and their relevance in nosocomial settings. The Infectious Diseases Society of America coined the acronym ESKAPE to emphasize these pathogens’ ability to evade common therapies through various drug resistance mechanisms [11]. These mechanisms include enzymatic inactivation, alteration of drug targets, changes in cell permeability (such as loss of porins or increased efflux pump expression), and protective mechanisms like biofilm formation [12]. The ESKAPE group includes a range of Gram-positive and Gram-negative species, including *Enterococcus faecium*, *Staphylococcus aureus*, *Klebsiella pneumoniae*, *Acinetobacter baumannii*, *Pseudomonas aeruginosa*, and *Enterobacter* spp. [12]. In a multicentre study involving 193 hospitals across the US, Marturano and Lowery found that 42.2% of the bacteria isolated from BSIs belonged to the ESKAPE group [13]. According to the latest European Centre for Disease Prevention and Control (ECDC) report on AMR surveillance, these pathogens continue to exhibit high levels of resistance to last-line antibiotics, as highlighted in previous reports from 2019. Increasing trends in resistance have been noted, particularly in carbapenem and third-generation-cephalosporin resistance among *Escherichia coli*, *Klebsiella pneumoniae*, *Pseudomonas aeruginosa*, and *Acinetobacter baumannii*, as well as vancomycin resistance in *Enterococcus faecium*, which has shown a significant rise. In Italy, data reported by Italian National Institute of Health, indicate a concerning increase in vancomycin resistance among *Enterococcus faecium* isolates, rising from 23.6% in 2020 to 28.2% in 2021 [14]. Therefore, continued vigilant monitoring of these trends is essential. In Europe, the prevalence of methicillin-resistant *Staphylococcus aureus* isolates (MRSA) has been declining. However, in Italy, after a stability period, there was an uptick in MRSA isolates in 2021. Additionally, over the past four years, in multiple European countries, including Italy, there has been a significant rise in the annual number of invasive *Acinetobacter baumannii* isolated [15,16]. Given the epidemiology of BSIs caused by ESKAPE pathogens and the clear limitations of current antimicrobial strategies, there is an urgent need to enhance empirical therapeutic approaches, while also developing new antimicrobial agents to combat the global health crisis associated with antibiotic resistance. The aim of this study was to evaluate the prevalence of BSIs caused by ESKAPE pathogens and to investigate their antimicrobial susceptibility patterns at San Giovanni di Dio e Ruggi d’Aragona University hospital, from 2020 to 2022.

## 2. Results

### 2.1. Prevalence of BSIs in Studied Patients

**Figure 1:**
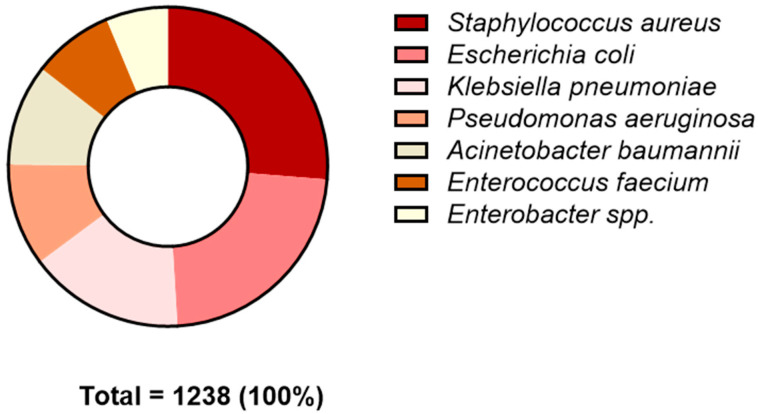
Prevalence of ESKAPE species from patients at San Giovanni di Dio and ‘‘Ruggi D’Aragona’’ University Hospital in Salerno from January 2020 to December 2022.

In the present study, blood cultures were examined to diagnose BSIs based on patients’ clinical symptoms, including: (i) fever (>38 °C), (ii) chills, (iii) hypotension, (iv) elevated white blood cell count, and (v) increased concentrations of inflammatory markers. Out of the blood cultures processed, 3197 tested positive. Among these, 1238 (38.7%) were positive for ESKAPE bacteria, and 1959 (61.3%) were positive for other pathogens (Table 1). Regarding gender distribution, of the 2087 BSI-positive patients, 837 (40.1%) and 1250 (59.9%) were from women and men, respectively. The difference between positive males and females was not statistically significant. Analyzing the age distribution of infections, the majority of positive cases were found in the elderly (≥60 years) (70.6%); followed by middle-aged adults (40–59 years) (19.5%); infants, children, and adolescents (0–19 years) 5%); and young adults (20–39 years) (Table 2). Our data indicate that the predominant bacterium isolated from blood samples was Staphylococcus aureus, with a prevalence of 26.3%. This was followed by *Escherichia coli* (22.8%), *Klebsiella pneumoniae* (15.8%), *Pseudomonas aeruginosa* (10.4%), *Acinetobacter baumannii* (10.3%), *Enterococcus faecium* (8.2%), and *Enterobacter* spp. (6.3%) (Figure 1).

### 2.2. Prevalence of Antimicrobial Resistance among Pathogens Identified in BSIs

The detailed antimicrobial resistance profiles of ESKAPE pathogens identified in our study are presented in Table 3 and Table 4. All isolated bacteria demonstrated a high level of resistance to the antibiotics tested. *Acinetobacter baumannii* exhibited resistance rates exceeding 78.8% to amikacin, ciprofloxacin, gentamicin, imipenem, meropenem, tobramycin, and trimethoprim/sulfamethoxazole. However, resistance to colistin was notably low, at less than 2.9%. *Escherichia coli* demonstrated a lower resistance rate of 63.5% to a range of antibiotics, including amikacin, amoxicillin/clavulanic acid, cefepime, ciprofloxacin, colistin, gentamicin, imipenem, meropenem, piperacillin/tazobactam, tobramycin, trimethoprim/sulfamethoxazole, ceftazidime, ceftazidime/avibactam, and ceftolozane/tazobactam. A significant decrease in gentamicin resistance was observed from 2020 to 2022, dropping from 31.5% to 19.4% (*p*-value = 0.046). Similarly, resistance to tobramycin significantly decreased from 32.3% to 19.2% during the same period (*p*-value = 0.032). In contrast, resistance to ceftazidime/avibactam notably increased, with resistant isolates rising from 0% in 2020 to 5.6% in 2022 (*p*-value = 0.020). Strains of *Klebsiella pneumoniae* exhibited resistance rates greater than 60% to various antibiotics, such as amoxicillin/clavulanic acid, cefepime, cefotaxime, ciprofloxacin, and piperacillin/tazobactam. Between 2020 and 2022, the prevalence of *Klebsiella pneumoniae* strains resistant to amikacin significantly decreased from 35.8% to 20.8% (*p*-value = 0.047). Resistance to ciprofloxacin also declined, with 82.4% of strains resistant in 2020 compared with 63.9% in 2022 (*p*-value = 0.020). Similarly, resistance to meropenem significantly dropped from 57.4% in 2020 to 36% in 2022. Resistance to colistin also showed a notable reduction, with colistin-resistant strains decreasing from 32.3% in 2020 to just 7% in 2022 (*p*-value < 0.001). Low antibiotic resistance rates were associated with *Pseudomonas aeruginosa* strains. In detail, a resistance rate of less than 44.7% was found for antibiotics including amikacin, cefepime, ceftazidime, ceftazidime/avibactam, ceftolozane/tazobactam, ciprofloxacin, colistin, imipenem, meropenem, tobramycin, and trimethoprim/sulfamethoxazole. Resistance to carbapenems (meropenem and imipenem) and tobramycin increased significantly from 2020 to 2022. Specifically, resistance to tobramycin rose from 12.5% in 2020 to 28.3% in 2022. For imipenem, resistance among *Pseudomonas aeruginosa* strains increased from 18.3% in 2020 to 34% in 2022. Similarly, resistance to meropenem escalated from 5.9% in 2020 to 32% in 2022. Among the Gram-positive strains, *Enterococcus faecium* demonstrated resistance rates exceeding 87.5% to penicillins and greater than 85.7% to quinolones. Resistance to glycopeptides was below 28.6%, while resistance to the sole active macrolide was less than 12.5%. Additionally, resistance to linezolid was less than 14.3%, and resistance to tigecycline was 6.3%. A significant increase in resistance to kanamicin was observed over the study period, with resistance rates rising from 81.3% in 2020 to 100% in 2022. Strains of Staphylococcus aureus exhibited resistance rates below 80.7% to a range of antibiotics, including trimethoprim/sulfamethoxazole, tigecycline, vancomycin, teicoplanin, tetracycline, rifampin, penicillin, oxacillin, mupirocin, linezolid, levofloxacin, daptomycin, erythromycin, gentamicin, and fosfomycin. Over the study period, levels of resistance to fosfomycin and vancomycin increased significantly. Throughout the study period, resistance to fosfomycin and vancomycin increased significantly. Specifically, resistance to fusidic acid rose from 2.4% in 2020 to 7.4% in 2022. Similarly, the prevalence of vancomycin-resistant strains increased from 1.2% in 2020 to 12.5% in 2022.

## 3. Discussion

BSIs pose an increasing threat to public health globally. In Europe, 2 million BSI cases annually result in 250,000 deaths, making BSIs the leading cause of infection-related mortality [17]. In our retrospective study, the number of positive blood cultures increased over the study period, with 820 in 2020, 1039 in 2021, and 1338 in 2022. The low number of blood cultures collected during the first wave of COVID-19 has also been reported in other studies [18,19]. This decrease can be attributed to a reduced access to healthcare departments and a decline in scheduled surgeries during the pandemic. Our data indicate that males were the most affected by BSIs, a finding also demonstrated by Uslan et al. In their study, nosocomial BSIs were more prevalent in males (23.8%) compared with females (13.9%), although this difference was not significant [20]. A prospective study conducted in Norway similarly found that men were at greater risk of experiencing a BSI than women [21]. This disparity can be attributed to physiological differences influencing susceptibility to infections, as well as extrinsic factors such as smoking, alcohol consumption, and recreational activities, which vary between sexes and contribute to the onset of blood infections. Furthermore, our study found that the age group most affected by BSIs was 60 to 79, representing more than 50% of cases. This is consistent with other studies, which have demonstrated that the incidence of bacteremia increases with age [20,22]. The increased incidence of MDR infections in older adults is primarily due to several age-related factors. Immune senescence significantly weakens the immune system, reducing both innate and adaptive immune responses. This leads to decreased production of naive T cells and diminished functionality of existing T and B cells, impairing the body’s ability to combat new and resistant pathogens. Additionally, older adults often have comorbidities such as diabetes, chronic obstructive pulmonary disease (COPD), and cardiovascular diseases, which further compromise their immune defenses. These conditions often necessitate medical interventions involving invasive devices like catheters or mechanical ventilators, which can increase infection risk. Anatomical and functional changes also contribute to increased vulnerability; for instance, thinning skin and mucosal membranes reduce physical barriers, while reduced lung function and bladder control lead to fluid stasis, promoting bacterial growth [23,24]. Moreover, older adults frequently encounter healthcare settings, which are hotspots for MDR organisms due to high antibiotic use and the presence of vulnerable populations. Frequent hospitalizations or stays in long-term care facilities further expose them to healthcare-associated infections (HAIs) caused by MDR pathogens. Collectively, these factors significantly elevate the risk of MDR infections in older adults [25]. BSIs caused by drug-resistant bacteria are linked to significant morbidity and mortality, extended hospital stays, and higher healthcare costs [26]. Among the most prevalent multidrug-resistant bacteria causing BSIs are the ESKAPE pathogens. In our study, ESKAPE pathogens were responsible for 38.7% of BSIs, while other pathogens accounted for 61.3% of cases. Within the ESKAPE group, *Staphylococcus aureus* was the most frequently isolated species from blood cultures, with a prevalence of 28%. This was followed by *Escherichia coli* (24.3%), *Klebsiella pneumoniae* (16.8%), *Pseudomonas aeruginosa* (11.1%), *Acinetobacter baumannii* (10.9%), and *Enterococcus faecium* (8.8%).

Consistent with our previous research, *Staphylococcus aureus* remains a leading cause of BSIs, with an average prevalence of 12.8% observed from 2015 to 2019 at San Giovanni di Dio and Ruggi D’Aragona Hospital. In line with the findings of Marturano et al., *Staphylococcus aureus* and *Escherichia coli* were the most prevalent ESKAPE pathogens in BSIs, comprising 21.9% and 22.6% of the total pathogens isolated from blood cultures, respectively [13]. In a study conducted in Iran, the prevalent species associated with BSIs were *Staphylococcus aureus* (30%), *Acinetobacter baumannii* (22%), *Pseudomonas aeruginosa* (17%), *Klebsiella pneumoniae* (13%), *Enterobacter aerogenes* (10.3%), and *Enterococcus faecium* (7.7%). The resistance patterns of the main ESKAPE strains have been reported. Our study revealed significant resistance patterns in *Staphylococcus aureus*, with high rates of resistance to erythromycin (48.1%), oxacillin (35.2%), and levofloxacin (34.4%). These findings are consistent with previous research by Licata et al. [27]. However, resistance to gentamicin decreased from 13.3% in 2015 to 5.7% in 2021, as noted by Santella et al. [28]. The COVID-19 pandemic has affected resistance trends, leading to increased resistance to linezolid, rifampicin, and teicoplanin, as shown by Golli et al. However, resistance to oxacillin decreased slightly from 36.1% in 2020 to 33.8% in 2022. Despite the stable or declining MRSA rates in several EU/EEA countries [16], resistance to teicoplanin increased at Ruggi D’Aragona Hospital. Notably, *Staphylococcus aureus* strains remain highly sensitive to vancomycin (92.7%) and daptomycin (94.2%), essential for treating MRSA infections. Antimicrobial resistance in *Enterococcus* species represents a significant global challenge due to their intrinsic resistance to multiple classes of antibiotics and limited effective therapeutic options. Our study detected vancomycin-resistant enterococci (VRE) in 21.2% of *E. faecium* strains. Resistance to vancomycin, mainly due to the vanA and vanB genes, is more common in *Enterococcus faecium* isolates. Epidemiological data from ECDC show a 33.2% increase in VRE strains in Europe from 2018 to 2022, with a slight decline of 4% from 2021 to 2022. Italy saw an increasing trend from 23.6% in 2020 to 30.7% in 2022, while countries such as France and Austria report lower resistance levels than Italy and Germany. Compared with previous data, our study was in line with Boccella et al., who reported an increase in vancomycin resistance in enterococci at Ruggi D’Aragona Hospital, from 3.7% to 18.75% for *Enterococcus faecium* [29]. Similarly, Lupia et al. found vancomycin resistance in 23.3% of *E. faecium* isolates. Despite the increase in VRE, our study showed that isolates remain highly sensitive to linezolid and tigecycline, although resistance to teicoplanin is almost as high as to vancomycin. Treatment for VRE BSIs has evolved, with recent evidence suggesting that high-dose daptomycin may reduce mortality more effectively than linezolid [30]. *Escherichia coli* is a major cause of BSIs in Europe, although it was the second most frequently isolated pathogen in our study. This bacterium represents a serious public health problem due to its reservoir of resistance genes. In 2022, we observed a marked increase in resistance among *Escherichia coli* isolates to third-generation cephalosporins, aminopenicillins, fluoroquinolones, and aminoglycosides, as reported by EARS-NET [31]. Despite this, carbapenem resistance remains low, with rates of 0.5% for imipenem and 1.1% for meropenem. The highest resistance rate was reported for third-generation cephalosporins (40.75%), followed by fluoroquinolones (36.4%), aminoglycosides (17%), and beta-lactam/beta-lactamase inhibitors (17%). These findings are consistent with Canadian studies linking resistance to increased mortality in *Escherichia coli*-related bacteremia [32]. The impact of COVID-19 on *Escherichia coli* infections has been mixed. An English study reported a decrease in *Escherichia coli* BSIs and resistance to piperacillin–tazobactam and ciprofloxacin in 2020 compared with 2019 [33]. In contrast, data from a New York hospital highlighted a slight increase in *Escherichia coli*-related bacteremia during the pandemic. The same trend was observed at the Ruggi D’Aragona Hospital, where *Escherichia coli* represented 10.71% of BSIs in 2019 and 8.9% in 2020 [28,34]. For *E. coli* infections resistant to third-generation cephalosporins, the AIFA guidelines recommend imipenem–cilastatin or meropenem as first-line treatments. Alternatives include cefepime and fosfomycin for AmpC-producing strains, or ceftazidime–avibactam and ceftolozane–tazobactam if other options are not suitable [35]. Our study found resistance rates to these alternative treatments of 2.9% and 6.3%, respectively. Antimicrobial resistance and virulence are critical factors influencing the pathogenicity of *Klebsiella pneumoniae*. This pathogen presents significant therapeutic challenges due to the limited efficacy of available antibiotics. In our study, *Klebsiella pneumoniae* isolates showed high rates of resistance to third-generation cephalosporins (72.9%), fluoroquinolones (68.9%), aminoglycosides (39.2%), and carbapenems (36.9%). These resistance patterns are consistent with global trends reported in the literature [36,37]. Resistance to third-generation cephalosporins, largely due to their widespread administration as first-choice empirical therapy, has a highly resonant global impact, affecting public health expenditure, and the spread of resistance by nosocomial infections with high death rates. Resistance to cephalosporins has led to an ever-increasing use of carbapenems over time, inducing resistance also for this class of molecules, leading to serious limitations in treatment options and representing a global call for the development of new antimicrobial molecules [14,38]. Carbapenem resistance showed an inconsistent trend during the study period. In 2020, resistance rates were approximately 42.5% for imipenem and 57.3% for meropenem. However, a notable decrease in resistance was observed in 2021, followed by a resurgence in 2022. This increase during the COVID-19 pandemic can be attributed to the excessive use of carbapenems to treat coinfected patients, as reported by Petrakis et al. [39]. In contrast, Italian data from 2020–2022 indicate a recent decline in carbapenem resistance among *Klebsiella pneumoniae* isolates. Given the complexity of treating carbapenem-resistant *Klebsiella pneumoniae* (CRE) infections, the use of ceftazidime/avibactam is recommended. Our study supports this approach, showing a high sensitivity of *Klebsiella pneumoniae* isolates to this combination of antibiotics (91.1%) [40]. An international cohort study conducted by EUROBACT 5 identified *Acinetobacter baumannii* as the most prevalent pathogen in BSIs in intensive care units (ICUs) [41]. Infections caused by *Acinetobacter baumannii* are associated with high mortality rates, with a 14-day mortality of 61.2% and a 30-day mortality of 73.6% reported in a study conducted in 12 Italian hospitals [42]. The multidrug resistance of the bacterium complicates treatment, leading to its classification by the WHO in 2018 as a “critical priority” pathogen due to carbapenem resistance. In our study, carbapenem resistance rates were alarmingly high, at 95% for imipenem and 95.5% for meropenem, consistent with other research but lower than EARS-NET surveillance data [31]. Resistance to aminoglycosides and fluoroquinolones also exceeded 80%, making colistin the most effective treatment, with a sensitivity rate of 97.5%. However, colistin resistance has been reported, with an overall rate of 4% and increasing prevalence from 2001 to 2023, as highlighted by a recent meta-analysis [43]. For the treatment of carbapenem-resistant *A. baumannii* BSI (CRAB), combination therapies such as ampicillin–sulbactam with minocycline or colistin are recommended [44]. EARS-NET data reveal that 32.4% of *Pseudomonas aeruginosa* isolates are resistant to at least one class of antibiotics (piperacillin–tazobactam, fluoroquinolones, ceftazidime, aminoglycosides, or carbapenems), with 19.7% resistant to two or multiple classes [31]. In Italy, resistance rates are highest for piperacillin–tazobactam (24.1%), followed by ceftazidime (19.0%), fluoroquinolones (18.5%), carbapenems (16.4%), and aminoglycosides (4%) [40]. Our study was in line with these findings, although fluoroquinolone resistance was higher, at 31.8%. During the study period, *Pseudomonas aeruginosa* retained significant susceptibility to colistin (93.3%) and amikacin (89.9%), with a notable decrease in amikacin resistance compared with the 2015–2019 period. Resistance to piperacillin–tazobactam remained stable, while resistance to ciprofloxacin and carbapenems showed a decline [45]. In contrast, a study conducted in southwestern China reported increased resistance to ciprofloxacin and gentamicin, but decreased resistance to piperacillin–tazobactam and imipenem during the pandemic [46]. This discrepancy in antimicrobial resistance and susceptibility profiles may be due to the different global localization and different therapeutic protocols implemented during the pandemic period. For BSIs from *Pseudomonas aeruginosa* with multidrug-resistant strains, ceftolozane–tazobactam, which showed a sensitivity of 86.9% in our study, is recommended. Resistance to ceftazidime–avibactam was found in 11.2% of isolates, while imipenem–relebactam was not tested at Ruggi D’Aragona Hospital.

## 4. Materials and Methods

### 4.1. Sample Collection

A blood samples were collected from patients at San Giovanni di Dio and ‘‘Ruggi D’Aragona’’ University Hospital in Salerno, from January 2020 to December 2022. The blood cultures were subsequently transported to the microbiology laboratory and processed.

### 4.2. Inclusion and Exclusion Criteria

The inclusion criteria were defined as follows: (i) enrolment of patients ranging in age from 0 to 99 years; (ii) inclusion of patients presenting with clinical and/or laboratory indicators indicative of bloodstream infection; and (iii) inclusion of patients from whom blood samples were collected specifically for requested microbiological analysis. Exclusion criteria were as follows: (i) patients with previous antimicrobial therapy that could have influenced antimicrobial susceptibility results; and (ii) positive blood cultures for non-ESKAPE-group pathogens.

### 4.3. Bacterial Culture and Identification

Blood cultures were incubated in the BD BACTEC™ Automated Blood Culture Monitoring System (Becton Dickinson Diagnostic Instrument Systems). The incubation protocol lasted 5 days, during which the system identified positive blood cultures. Subsequently, one drop from each flask was plated onto agar media, including chocolate agar, CNA blood agar, MacConkey agar, and Sabouraud Glucose agar (Oxoid, Hampshire, UK) for the detection of aerobic pathogens. In cases of positivity for anaerobic bacteria, chocolate agar and Schaedler Blood Agar media were used. All plates were incubated overnight at 37 °C under aerobic conditions, except for chocolate agar plates, which were incubated in 5% CO_2_, and Schaedler Blood Agar medium, which was incubated under anaerobic conditions. Bacterial identification was achieved using matrix-assisted laser desorption/ionization time-of- flight mass spectrometry (MALDI-TOF MS) (bioMérieux, Marcy l’Etoile, France). Two colonies from an agar plate culture were transferred to VITEK MS-DS slide (bioMérieux). Each well was coated with 1 μL of matrix solution (VITEK MS-CHCA, bioMérieux, Marcy l’Etoile, France), a saturated solution of alpha-cyano-4-hydroxycinnamic acid, and dried for 5 min. The resulting spectra were imported into MYLA (bioMérieux, Marcy l’Etoile, France) and analyzed using standard pattern matching against reference spectra. The percentage probability range of between 60 and 99% showed a good level of identification.

### 4.4. Antibiotic Susceptibility Assays

The VITEK2 system (bioMérieux, Marcy l’Etoile, France) was utilized for confirming species identification obtained via MALDI-TOF MS and for conducting antibiotic susceptibility testing. Pure bacterial colonies were inoculated into a test tube containing 3 mL of a 0.45% sodium chloride solution. A Densichek (bioMérieux, Marcy l’Etoile, France) was used to adjust the bacterial suspension to a McFarland standard of 0.5. The identification cards (ID-GN for Gram-negative bacteria and ID-GP for Gram-positive bacteria) were placed in the tubes containing the inoculum, while the sensitivity cards (AST-658, AST-659, and AST-397) were inserted inside the empty test tubes. The results of antimicrobial susceptibility tests were interpreted according to EUCAST guidelines. The following antimicrobials were assessed in this study: amoxicillin/clavulanic acid, cefepime, cefotaxime, ceftazidime, ciprofloxacin, colistin, imipenem, meropenem, gentamicin, levofloxacin, kanamicin high level, oxacillin, and streptomycin.

### 4.5. Data Analysis

The Cochrane–Armitage test was employed to evaluate the statistical significance of trends. XLSTAT was used to conduct statistical analysis (Lumivero (2024). XLSTAT statistical and data analysis solution. Paris, France). An alpha level of 0.05 was considered significant. Ethical approval was waived for this retrospective analysis of anonymized data.

## 5. Conclusions

Antimicrobial resistance represents a silent pandemic poised to cause more deaths than cancer. This study evaluated the resistance patterns of ESKAPE pathogens responsible for BSIs. The primary pathogens involved were Staphylococcus aureus and *Escherichia coli*, with prevalences of 26.3% and 22.8%, respectively. Unfortunately, the mismanagement of antibiotics has not only led to increased resistance to common antibiotics, but also compromised the efficacy of newer drugs. For instance, Staphylococcus aureus exhibited a significant rise in resistance to fosfomycin and vancomycin between 2020 and 2022. Similarly, *Escherichia coli* showed increased resistance to the latest-generation antibiotic ceftazidime/avibactam, highlighting the consequences of poor management of new antibiotics. The significant rise in resistance to both common and newer antibiotics underscores the urgent need for improved antibiotic stewardship. Addressing this issue is critical to preserving the efficacy of existing treatments and preventing a future dominated by untreatable infections.

## Figures and Tables

**Table 1:** BSI distribution of ESKAPE and non-ESKAPE pathogens among positive blood cultures.

Pathogen	N (%)
Pathogenic bacteria	3197
ESKAPE pathogen	1238 (38.7)
Other pathogen	1959 (61.3)

**Table 2:** Gender and age distribution in patients with positive blood cultures.

Gender	2020	2021	2022	TOT	%
**Female**	225	295	317	837	40.1
**Male**	348	441	461	1250	59.9
**Total**	573	736	778	2087	
**Age (years)**	2020	2021	2022	TOT	%
**0–19**	33	38	33	104	5.0
**20–39**	39	35	29	103	4.9
**40–59**	129	131	146	406	19.5
**≥** **60**	372	532	408	1474	70.6
**Total**	573	736	778	2087	

**Table 3:** Resistant ESKAPE Gram-negative strains isolated from blood cultures.

***Acinetobacter baumannii***	**2020 (R%)**	**2021**	**2022**	***p*-Value**
Amikacin	78.8 (41)	88.9 (32)	87.5 (35)	0.233
Ciprofloxacin	98.0 (50)	94.4 (34)	94.9 (37)	0.422
Colistin	2.0 (1)	2.9 (1)	2.8 (1)	0.820
Gentamicin	94.1 (48)	94.4 (34)	89.7 (35)	0.444
Imipenem	95.7 (44)	94.4 (34)	94.9 (37)	0.862
Meropenem	96.1 (49)	100 (19)	92.5 (37)	0.448
Tobramycin	93.3 (42)	94.3 (33)	88.9 (32)	0.479
Trimethoprim/sulfam	94.1 (48)	91.7 (33)	87.2 (34)	0.252
***Escherichia coli***	**2020**	**2021**	**2022**	***p*-Value**
Amikacin	0 (0)	1.3 (1)	1.6 (2)	0.322
Amoxicillin/clav. acid	46.7 (28)	49.3 (36)	41.9 (54)	0.435
Cefepime	33.9 (21)	36.3 (29)	30.2 (39)	0.519
Cefotaxime	46.6 (34)	37.0 (17)	43.4 (56)	0.751
Ceftazidime	39.7 (29)	45.0 (36)	34.1 (44)	0.323
Ceftazidime/avibactam	0 (0)	0.0 (0)	5.6 (7)	0.020
Ceftolozane/tazobactam	3.8 (2)	6.6 (4)	7.2 (9)	0.413
Ciprofloxacin	63.5 (47)	57.5 (46)	51.9 (67)	0.107
Colistin	0 (0)	0.0 (0)	3.2 (4)	0.061
Gentamicin	31.5 (23)	27.5 (22)	19.4 (25)	0.046
Imipenem	0 (0)	0.0 (0)	1.6 (2)	0.180
Meropenem	0 (0)	1.7 (1)	1.5 (2)	0.359
Piperacillin/tazobactam	10.8 (8)	16.0 (13)	10.1 (13)	0.725
Tobramycin	32.3 (20)	32.4 (22)	19.2 (24)	0.032
Trimethoprim/sulfam	47.3 (35)	37.5 (30)	47.3 (61)	0.826
***Klebsiella pneumoniae***	**2020**	**2021**	**2022**	***p*-Value**
Amikacin	35.8 (24)	21.4 (12)	20.8 (15)	0.047
Amoxicillin/clav. acid	85.0 (51)	69.6 (39)	80.6 (58)	0.602
Cefepime	74.5 (35)	48.2 (27)	65.3 (47)	0.493
Cefotaxime	83.8 (57)	65.2 (30)	69.4 (50)	0.056
Ceftazidime	82.4 (56)	61.4 (35)	70.8 (51)	0.137
Ceftazidime/avibactam	11.4 (5)	5.6 (3)	9.9 (7)	0.895
Ceftolozane/tazobactam	45.5 (20)	49.1 (26)	47.9 (34)	0.828
Ciprofloxacin	82.4 (56)	58.9 (33)	63.9 (46)	0.020
Colistin	32.3 (21)	3.8 (2)	7.0 (5)	<0.001
Gentamicin	43.3 (29)	17.9 (10)	33.3 (24)	0.230
Imipenem	42.6 (20)	24.1 (14)	33.3 (24)	0.406
Meropenem	57.4 (39)	24.5 (12)	36.0 (27)	0.011
Piperacillin/tazobactam	67.6 (46)	63.2 (36)	73.6 (53)	0.438
Tobramycin	63.6 (28)	38.5 (20)	43.7 (31)	0.063
Trimethoprim/sulfam	45.6 (31)	48.2 (27)	47.2 (34)	0.849
***Pseudomonas aeruginosa***	**2020**	**2021**	**2022**	***p*-Value**
Amikacin	8.8 (3)	4.2 (2)	17.0 (8)	0.170
Cefepime	26.9 (7)	12.2 (6)	36.2 (17)	0.235
Ceftazidime	35.3 (12)	18.8 (9)	36.2 (17)	0.774
Ceftazidime/avibactam	13.6 (3)	7.7 (3)	13.0 (6)	0.902
Ceftolozane/tazobactam	22.7 (5)	7.7 (3)	13.0 (6)	0.423
Ciprofloxacin	20.6 (7)	33.3 (16)	38.3 (18)	0.098
Colistin	10.0 (3)	2.3 (1)	8.7 (4)	0.975
Imipenem	18.5 (5)	16.3 (8)	34.0 (16)	0.078
Meropenem	5.9 (2)	21.4 (9)	32.0 (16)	0.004
Piperacillin/tazobactam	39.4 (13)	29.2 (14)	44.7 (21)	0.521
Tobramycin	12.5 (3)	9.1 (4)	28.3 (13)	0.046

Data are reported as resistance percentages (number of resistant isolates).

**Table 4:** Resistant ESKAPE Gram-positive strains isolated from blood cultures.

***Enterococcus faecium***	**2020**	**2021**	**2022**	***p*-Value**
Amoxicillin/clav. acid	93.3 (14)	100 (32)	98.0 (48)	0.491
Ampicillin	90.0 (18)	97.0 (32)	98.0 (48)	0.158
Ampicillin/sulbactam	87.5 (7)	90.9 (20)	95.9 (47)	0.466
Quinupristin/dalfopristin	12.5 (2)	6.1 (2)	2.0 (1)	0.097
Ciprofloxacin	85.7 (6)	100 (22)	95.9 (47)	0.580
Imipenem	90.0 (18)	100 (33)	98.0 (48)	0.159
Kanamycin high level	81.3 (13)	84.4 (27)	100 (49)	0.005
Levofloxacin	85.7 (6)	100 (22)	98.0 (48)	0.238
Linezolid	14.3 (3)	5.9 (2)	4.1 (2)	0.145
Streptomycin high level	75.0 (12)	84.4 (27)	89.6 (43)	0.155
Teicoplanin	28.6 (6)	23.5 (8)	18.8 (9)	0.355
Tigecycline	6.3 (1)	6.3 (2)	6.3 (3)	0.997
Vancomycin	23.8 (5)	22.9 (8)	18.8 (9)	0.595
***Staphylococcus aureus***	**2020**	**2021**	**2022**	***p*-Value**
Fusidic acid	2.4 (2)	1.9 (2)	7.4 (10)	0.053
Ceftaroline	1.3 (1)	0.9 (1)	1.5 (1)	0.871
Daptomycin	4.8 (4)	2.8 (3)	8.8 (12)	0.146
Erythromycin	44.6 (37)	51.0 (53)	48.2 (66)	0.679
Gentamicin	6.0 (5)	5.7 (6)	6.6 (9)	0.844
Levofloxacin	35.7 (30)	33.0 (35)	34.6 (47)	0.900
Linezolid	0 (0)	0 (0)	2.2 (3)	0.070
Mupirocin	0 (0)	1.9 (2)	0.7 (1)	0.748
Oxacillin	36.1 (30)	36.2 (38)	33.8 (46)	0.702
Benzylpenicillin	80.7 (67)	72.0 (77)	69.9 (95)	0.091
Rifampicin	6.0 (5)	4.8 (5)	7.4 (10)	0.620
Teicoplanin	9.6 (8)	10.4 (11)	11.7 (16)	0.624
Tetracycline	7.2 (6)	2.9 (3)	2.9 (4)	0.147
Tigecycline	0 (0)	0 (0)	0.7 (1)	0.296
Trimethoprim/sulfam	1.2 (1)	3.8 (4)	3.7 (5)	0.339
Vancomycin	1.2 (1)	5.5 (6)	12.5 (17)	0.001

Data are reported as resistance percentages (number of resistant isolates).

## Data Availability

Data are contained within the article.
